# Enhancing diabetic muscle repair through W-GA nanodots: a nanomedicinal approach to ameliorate myopathy in type 2 diabetes

**DOI:** 10.1093/burnst/tkae059

**Published:** 2025-01-24

**Authors:** Shan Liu, Renwen Wan, QingRong Li, Yisheng Chen, Yanwei He, Xingting Feng, Patrick Shu-Hang Yung, Zhiwen Luo, Xianwen Wang, Chen Chen

**Affiliations:** Department of Endocrinology, Huashan Hospital, Fudan University, No. 12. Middle Wulumuqi Road, Jingan District, Shanghai 20040, China; Department of Sports Medicine, Huashan Hospital, Fudan University, No. 12. Middle Wulumuqi Road, Jingan District, Shanghai 200040, China; School of Biomedical Engineering, No. 81 Meishan Road, Shushan District, Anhui Medical University, Hefei 230032, China; Department of Sports Medicine, Huashan Hospital, Fudan University, No. 12. Middle Wulumuqi Road, Jingan District, Shanghai 200040, China; Department of Sports Medicine, Huashan Hospital, Fudan University, No. 12. Middle Wulumuqi Road, Jingan District, Shanghai 200040, China; Department of Sports Medicine, Huashan Hospital, Fudan University, No. 12. Middle Wulumuqi Road, Jingan District, Shanghai 200040, China; Department of Orthopaedics and Traumatology, Faculty of Medicine, The Chinese University of Hong Kong, Shatin 999077, Hong Kong; Department of Sports Medicine, Huashan Hospital, Fudan University, No. 12. Middle Wulumuqi Road, Jingan District, Shanghai 200040, China; School of Biomedical Engineering, No. 81 Meishan Road, Shushan District, Anhui Medical University, Hefei 230032, China; Department of Arthroscopic Surgery, Shanghai Jiao Tong University Affiliated Sixth People's Hospital, No. 600 Yishan Road, Xuhui District, Shanghai 200233, China

**Keywords:** Type 2 diabetes, Muscle injury, Nanodots, Muscle regeneration

## Abstract

**Objective:**

Type 2 diabetes mellitus (T2DM) is a chronic metabolic disorder that significantly impairs muscle regeneration following injuries, contributing to numerous complications and reduced quality of life. There is an urgent need for therapeutic strategies that can enhance muscle regeneration and alleviate these pathological mechanisms. In this study, we evaluate the therapeutic efficacy of W-GA nanodots, which are composed of gallic acid (GA) and tungstate (W6+), on muscle regeneration in type 2 diabetes mellitus (T2D)-induced muscle injury, with a focus on their anti-inflammatory and antioxidative effects.

**Methods:**

This study synthesized ultrasmall W-GA nanodots that were optimized for improved stability and bioactivity under physiological conditions. In vitro assessments included cell viability, apoptosis, reactive oxygen species (ROS) generation, and myotube differentiation in C2C12 myoblasts under hyperglycemic conditions. In vivo, T2D was induced in C57BL/6 mice, followed by muscle injury and treatment with W-GA. Muscle repair, fibrosis, and functional recovery were assessed through histological analysis and gait analysis using the CatWalk system.

**Results:**

The W-GA nanodots significantly enhanced muscle cell proliferation, decreased ROS, and reduced apoptosis in vitro. In vivo, compared with the control group, the W-GA-treated group exhibited notably improved muscle regeneration, decreased fibrosis, and enhanced functional recovery. The treatment notably modulated the inflammatory response and oxidative stress in diabetic muscle tissues, facilitating improved regenerative dynamics and muscle function.

**Conclusions:**

W-GA nanodots effectively counter the pathological mechanisms of diabetic myopathy by enhancing regenerative capacity and reducing oxidative stress and inflammation. This nanomedicine approach offers a promising therapeutic avenue for improving muscle health and overall quality of life in individuals suffering from T2D. However, further studies are needed to explore the clinical applications and long-term efficacy of these nanodots in preventing diabetic complications.

## Background

The global epidemic of obesity and its associated chronic metabolic disorders, particularly type 2 diabetes (T2D), poses a formidable challenge to public health systems worldwide [1–3]. Insulin resistance and pancreatic β-cell dysfunction are the primary etiologies of T2D, a condition characterized by a high global prevalence and associated with numerous clinical complications [4–6]. Skeletal muscle plays a critical role in postprandial glucose uptake in healthy individuals, and impairments in glucose sensing and utilization within this tissue are common hallmarks of the progression of metabolic diseases related to obesity [1, 7, 8]. The heterogeneity and plasticity in the metabolic and contractile functions of skeletal muscle fibers significantly influence muscle health and disease [9–11].

Over the past few decades, multiple mechanisms underlying the pathogenesis of T2D have been elucidated, including excessive intramuscular and intermuscular fat accumulation, decreased mitochondrial oxidative capacity leading to oxidative stress, and a shift from oxidative to glycolytic muscle fibers [12–14]. Collectively, these factors contribute to the deterioration of muscle metabolism and function. Notably, obesity and T2D impair not only muscle insulin sensitivity and glucose metabolism but also postinjury muscle regeneration, leading to a metabolic syndrome known as diabetic myopathy. This syndrome is characterized by a progressive decline in metabolic activity, muscle strength, and mass, resulting in impaired muscle repair following injury [15, 16]. Furthermore, these muscle complications are believed to exacerbate systemic energy homeostasis, thereby promoting the progression of T2D and its associated complications, such as cardiovascular diseases and nonalcoholic fatty liver disease [17, 18].

Despite their significant clinical implications, the issues of impaired postinjury muscle regeneration and the inhibition of myopathy due to obesity and T2D have not received adequate attention in clinical practice. Current strategies to address these challenges include pharmacological interventions, lifestyle modifications, and physical therapy; however, these approaches often fall short in enhancing muscle regeneration and reducing chronic inflammation [19–21]. There remains an urgent need for more effective therapeutic approaches.

Muscle stem cells (MSCs), located between the basement membrane and the plasma membrane of muscle fibers, typically remain quiescent under normal physiological conditions [22–24]. Upon injury or external stimuli, these cells exhibit potent regenerative capabilities and play a crucial role in muscle maintenance and plasticity. The process of muscle repair involves a highly coordinated sequence of muscle degeneration, inflammation, regeneration, and remodeling [25, 26]. However, in muscle injuries associated with T2D, this regenerative potential is often suppressed, resulting in prolonged muscle damage.

Raw materials, including gallic acid (GA) and tungstate (W6+), were selected for their distinct properties. Gallic acid, a polyphenolic organic compound, is well known for its potent antioxidative and anti-inflammatory properties [27–29]. Tungstate, a multifunctional metal, has demonstrated therapeutic potential by inhibiting inflammatory responses [30–32]. These materials form the foundation of our nanodots, which are designed to enhance muscle regeneration and function. In response to the urgent need for effective treatments for diabetic muscle injuries, our study focused on the synthesis of ultrasmall coordination polymer nanodots incorporating GA and W6+ [32, 33]. These nanodots, composed of a polyphenolic organic compound and a multifunctional metal, are designed to mitigate muscle injuries through their anti-inflammatory and antioxidative properties while promoting MSC viability [34, 35]. However, the therapeutic efficacy of GA and other natural antioxidants in modulating the muscle injury microenvironment is constrained by their instability under harsh conditions, poor pharmacokinetics, high water solubility, potential toxicity at high doses, and nonspecific tissue accumulation. To overcome these limitations and develop a fully functional nanomedicine, we aimed to synergize the properties of GA with other natural products. Our investigations revealed that nanodots modified with polyvinylpyrrolidone (PVP) and coordinated with tungsten and GA demonstrate exceptional capacity for scavenging nitrogen and oxygen radicals, exhibiting superoxide dismutase (SOD) mimetic activity that contributes to the alleviation of inflammatory symptoms.

Thus, our study synthesized and applied W-GA nanodots for the repair of muscle injuries in T2D patients. Through in vitro experiments, we validated their ability to modulate the immune microenvironment, promote myotube formation, and facilitate functional recovery. Moreover, comprehensive in vivo evaluations, including detailed histological and behavioral assessments, demonstrated that these multifunctional W-GA nanodots are biologically active materials suitable for the repair of T2D muscle injuries, offering a novel and promising strategy for the clinical treatment of diabetic skeletal muscle injuries.

## Methods

### Preparation of W-GA nanodots

According to previous literature [32], 80 mg of WCl_6_ was added to 16 mL of water and vigorously stirred to prepare a WCl6 solution. Subsequently, a polyvinylpyrrolidone (PVP) solution (66 mg/mL, 2 mL) was added to the WCl_6_ solution and stirred for one hour. Then, 2 mL of a gallic acid solution (10 mg/mL) was slowly added, and the mixture was allowed to react for 12 hours. The resulting solution was dialyzed against deionized water for 24 hours using a dialysis membrane with a molecular weight range of 8000–14 000 Da. Finally, the ultrasmall W-GA nanodots were quantified using ICP–MS and stored at 4°C for future use.

### Characterization of W-GA nanodots

UV–visible absorption spectra were acquired using a Genesys 50 spectrophotometer (Thermo Scientific, China). Optical densities were measured using a Universal Microplate Spectrophotometer, specifically the Synergy2 SLFPTAD model (USA). X-ray diffraction profiles were acquired using a Rigaku SmartLab SE X-ray diffractometer (Japan). Sample morphology was characterized using a JEM-1400 Plus transmission electron microscope (Japan). Chemical analysis and state determination were conducted using X-ray photoelectron spectroscopy (XPS) with a K-Alpha instrument from Thermo Scientific in China. Fluorescence imaging was performed using a U-LH100HGAPO fluorescence microscope (Olympus Corporation, Japan).

### Cell culture

C2C12 myoblasts (a mouse cell line) were obtained from ScienCell Research Laboratories and cultured in Dulbecco's modified Eagle’s medium (DMEM; containing 25 mM glucose; Gibco, USA), which included 10% fetal bovine serum (Gibco, USA), 100 U/mL penicillin, and 100 μg/mL streptomycin (Gibco, USA), in a humidified atmosphere at 37°C and 5% CO_2_. Upon the cells reached 90% confluence, the medium was replaced with DMEM containing 2% horse serum (Gibco, USA), 60 mM D-glucose was added to simulate an insulin-resistant environment for induced differentiation, and 200 μg/mL W-GA or an equivalent volume of PBS was added to the respective groups [36–38].

### Live/dead cell staining

The effects of W-GA nanodots on cell viability were evaluated using a live/dead cell staining kit (Beyotime, China) [39]. C2C12 cells were seeded in a 24-well plate (5 × 10^4^ cells per well) and incubated for 24 hours. Subsequently, the medium was supplemented with 200 μg/mL W-GA nanodots or an equivalent volume of PBS, and incubation was continued. After an additional 24 hours, the residual culture medium was removed, and the cells were washed twice with PBS. Subsequently, 300 μL of PBS containing calcein AM and propidium iodide was added to each well, followed by incubation at 37°C for 30 minutes. Live cells exhibiting bright green fluorescence and dead cells exhibiting deep red fluorescence were then observed using a confocal microscope (Nikon, Japan).

### C‌CK-8 assay

The viability of C2C12 cells post-treatment with W-GA nanodots was assessed using a Cell Counting Kit-8 (CCK-8, Beyotime, China). The experimental protocol was designed based on methodologies established in prior studies [40]. Briefly, TDSCs were seeded at a density of 1 × 10^3^ cells per well in a 96-well plate (Corning, USA) and subjected to various treatments for 24 hours. Afterward, 10 μL of CCK-8 solution was added to each well, and the plates were incubated for an additional 2 hours. The optical density at a wavelength of 450 nm was measured using a microplate reader (Bio-Rad, USA). The cell viability was calculated as the ratio of the average optical density of the treated samples to that of the control samples multiplied by 100%.

### BrdU

The proliferation of C2C12 cells was evaluated using a 5-bromo-2′-deoxyuridine (BrdU) incorporation kit from Cell Signaling Technology (USA) according to the manufacturer's guidelines. Briefly, C2C12 cells were seeded at a density of 1 × 10^6^ cells per well in a 6-well plate and subjected to various treatments for 24 hours. Subsequently, 12 hours post-treatment, BrdU solution was added to each well. Thereafter, the cells were fixed, washed twice with phosphate-buffered saline (PBS), and incubated with a rat anti-BrdU primary antibody at room temperature for one hour. Finally, the nuclei were restained with 4′,6-diamidino-2-phenylindole (DAPI) for five minutes. Cell proliferation was quantified using the following formula: the average number of BrdU-positive cells divided by the total number of DAPI-stained cells, multiplied by 100% [41].

### Flow cytometry analysis

Flow cytometry analysis (FACS) primarily serves as a tool for identifying cell apoptosis and detecting reactive oxygen species (ROS). The specific methods used for these analyses will be described in subsequent sections. Briefly, cells subjected to various treatment protocols were meticulously labeled using multiple kits or flow cytometry antibodies. Subsequently, cell apoptosis and ROS scavenging were detected using FACS (BD, USA). Finally, the data obtained were analyzed using FlowJo software (version 10).

### Apoptosis assay

We evaluated apoptosis using the Annexin V-FITC Apoptosis Detection Kit (Beyotime, China). To assess the antiapoptotic potential of W-GA nanodots, C2C12 cells were seeded at 1 × 10^6^ cells per well in a 6-well plate and incubated for 24 hours to ensure optimal attachment and growth. Following this incubation, the medium was replaced with 200 μg/mL W-GA nanodots or an equivalent volume of PBS, and treatment continued for 6 hours. After treatment, the cells were gently detached with trypsin, washed carefully twice with phosphate-buffered saline (PBS), and then resuspended in Annexin V binding buffer. Subsequently, Annexin V and propidium iodide (PI) were added to the cell suspension, which was incubated at room temperature for 15 minutes in the dark to achieve appropriate staining. Ultimately, cell samples were analyzed using a flow cytometer (BD, USA), revealing crucial insights associated with apoptotic events.

### In vitro reactive oxygen species detection

To evaluate the scavenging efficiency of hydrogels on oxygen radicals, C2C12 cells were plated at a density of 1 × 10^6^ cells per well in a six-well plate and incubated for 24 hours to ensure normal adherent growth. Subsequently, the medium was replaced with 200 μg/mL W-GA nanodots or an equivalent amount of PBS, and the cells were incubated for 6 hours. Afterward, staining was performed using 2′,7′-dichlorofluorescein diacetate (DCFH-DA; Beyotime, China), and the intensity of ROS in the FITC channel was accurately quantified using a flow cytometer (BD, USA).

### Animals

All animal experiments were conducted according to the guidelines of the National Institutes of Health (NIH) Guide for the Care and Use of Laboratory Animals. The Laboratory Animal Ethics Committee of the First Affiliated Hospital of the University of Science and Technology of China approved the experimental protocol (Approval No: 2022-N(A)-061), with all efforts made to minimize animal suffering. In this study, a total of 75 male C57BL/6 mice (aged 6–8 weeks; weight: 20 ± 3 g) were obtained from the Shanghai Laboratory Animal Center and housed in the animal care facility of the Department of Laboratory Animal Science at Fudan University. The mice were maintained under controlled temperature, humidity, and lighting conditions, with free access to rodent chow and water, and a 12-hour light/dark cycle was used.

### Establishment of the T2DM model

After 7 days of acclimation, C57 mice were randomly divided based on body mass into two groups: a control group (*n* = 10, fed a normal caloric diet) and a type 2 diabetes mellitus model group (*n* = 70, fed a high-fat diet) with dietary induction for 12 weeks. After a 12-hour fast with access to water, the model group mice received an intraperitoneal injection of STZ at a dose of 30 mg/(kg·d) for five consecutive days. STZ was administered to destroy pancreatic β-cells to induce type 2 diabetes mellitus in the mice. Similarly, the control group was injected with 0.01 M sodium citrate buffer (pH 4.5) [42].

### Glucose tolerance test

Diabetes induction was verified eight days after the last dose of STZ using the intraperitoneal glucose (2 g/kg) tolerance test (IPGTT). After bedding was replaced, the mice were fasted overnight for approximately 16 hours, after which their fasting body weights were recorded the next day. Tail tips are clipped to sample blood for fasting glucose measurements. The appropriate intervals for injections were determined based on the number of mice. The first mouse was injected intraperitoneally with glucose solution. Fifteen minutes later, blood was sampled from the tail tip of the first mouse to assess blood glucose levels 15 minutes postglucose load. Following the method described above, blood glucose levels were measured at 30, 60, and 120 minutes. Throughout the experiment, the mice were gently handled, and a relatively quiet environment was maintained to prevent stress-induced increases in blood glucose levels. Blood glucose levels were measured with a Roche glucometer [43].

### Establishment of the mouse contusion model

The successfully generated T2DM model mice were randomly divided into four groups (15 mice each): (i) Control group (no treatment), (ii) contusion group, (iii) contusion + PBS group, (iv) I Contusion+W-GA group. Acute muscle contusion was performed on the right gastrocnemius muscle (GM) of all mice, as described in our previous study [44]. Briefly, after anesthesia with 1% pentobarbital sodium (0.5 mL/100 g) via intraperitoneal injection, the right hindlimbs of the mice were fixed on a platform with the GM exposed. Subsequently, a 20 g, 2-cm stainless steel ball was dropped from a height of 1 meter to impact the GM. The mice were then allocated to different treatment groups: in the Contusion+W-GA group, 10 mg/kg W-GA nanodots were injected proximally and distally into the GM; in the Contusion+PBS group, an equivalent volume of PBS solution was injected at similar locations. At 3, 7, and 14 days postinjury, samples were collected from the right GM of 5 mice in each group to assess muscle fibrosis and functional recovery.

### Single-cell RNA-seq using 10x genomics chromium

Samples from 10 experimentally treated mice were pooled, and scRNA-seq was performed on all surviving cells isolated from the gastrocnemius muscle. The gastrocnemius muscle was digested for 30 minutes with 0.15 g/100 mL of collagenase I and filtered through a 40-μm cell strainer. Single isolated cells were treated with red blood cell lysis buffer and subjected to magnetic bead separation to remove red and dead cells. The viability of single cells postfiltering was assessed via trypan blue staining (Thermo Fisher Scientific, Waltham, MA, USA) and using a hemocytometer (Thermo Fisher Scientific). Following counting, the volume was calculated based on the capture of 7000 cells. Samples with cell concentrations below the recommended 700–1200 cells/μL were pelleted, resuspended in a reduced volume, and recounted using a hemocytometer before loading onto the 10x Genomics single-cell A-chip. Reverse transcription and library preparation were carried out using the 10x Genomics Single Cell v2 kit in accordance with the 10x Genomics protocol. Libraries were multiplexed and sequenced on a single channel of the Illumina NextSeq-500 platform using a high-output (400 m) kit. Quality control checks were conducted for the 10x Genomics single-cell RNA-seq data. Sequences obtained from the 10x Genomics single-cell RNA-seq platform were demultiplexed and mapped to the mm10 transcriptome using Cell Ranger software (10x Genomics). Subsequently, raw digital gene expression matrices (UMI counts per cell per gene) analyzed with Cell Ranger software were filtered and normalized using the R package Seurat (version 2.3.4) [45]. Cells with fewer than 500 unique genes expressed, UMI counts greater than 50 000, or mitochondrial reads exceeding 10% were removed. Genes not detected in any cells were removed from further analysis [46].

### Processing of single-cell RNA sequencing data

Logarithmic normalization and linear regression were performed using the NormalizeData and ScaleData functions in Seurat to generate gene expression matrices. Principal component analysis was performed using the RunPCA function in Seurat for dimensionality reduction clustering. DecontX is used to predict contamination levels and to remove aberrantly expressed marker genes in mixed datasets. Harmony was applied to eliminate batch effects at the sample level. The cell subpopulations were visualized with UMAP, and feature gene expression was evaluated using Seurat software (Dotplot, FeaturePlot, and other functions). Differential expression analysis was conducted using the “FindAllMarkers” function in Seurat, utilizing a likelihood ratio test assuming that the data followed a negative binomial distribution and considering only genes in >25% of cells in the cluster with a fold change greater than log2(0.25). Cell types were identified using “Single R” software, published research, and expert experience [47].

### Pathway enrichment analysis

Functional enrichment analysis, including Gene Ontology (GO) and Kyoto Encyclopedia of Genes and Genomes (KEGG), was used to determine which differentially expressed genes (DEGs) were significantly enriched in GO terms or metabolic pathways. GO is an international standard gene function classification system. Differentially expressed genes (DEGs) were mapped to GO terms representing biological functions within the database. The number of genes within each term was calculated, and a hypergeometric test was performed to identify GO terms significantly enriched in the gene list beyond the background of the reference gene list. GO terms and KEGG pathways with a false discovery rate (FDR) of *P* < 0.05 were deemed significantly enriched [35].

### Gait analyses

The gait performance of the mice was meticulously assessed using the CatWalk XT gait analysis system (CatWalk XT; Noldus, the Netherlands) [48]. For the Contusion and Treatment groups, surgical intervention was limited to the right hind limb, with the left hind limb unaffected. This approach aims to prevent potential changes in mouse posture to maintain balance. Such posture adjustments could diminish the discernment of differences between the two hind limbs. Conversely, mice in the normal group did not undergo any surgical intervention on any hind limb. To familiarize the mice with the task at hand, a rigorous training regimen was implemented, requiring the mice to traverse from one end of an enclosed corridor to the other, along an illuminated glass panel. The focus was on continuous movement without any prolonged stops, which lasted at least one week. The footprints produced by the mice were recorded using internal light footprint refraction technology. Each footprint not only displayed its area but also provided a visual representation of the relative pressure exerted by the foot. The intensity of the emitted light was proportional to the magnitude of the relative pressure. A high-speed camera was used to precisely record the footprints, which were then analyzed automatically using CatWalk XT 10.0 software.

### Histological analysis

At 3, 7, and 14 days postcontusion, each mouse's right gastrocnemius muscle (GM) was carefully excised and fixed in a % paraformaldehyde (PFA) solution for 24 hours. After 24 hours, the muscle samples were dehydrated sequentially in 70%, 80% (twice), 95% (twice), and 100% (three times) ethanol, and each step lasted 30 minutes. Subsequently, the samples were subjected to three 20-minute treatments with xylene and embedded in paraffin. Next, the embedded injured muscle tissue was sectioned into 5-μm thick slices (perpendicular to the muscle fibers) using a Leica RM2235 paraffin microtome for routine hematoxylin and eosin (H&E) staining, Masson's trichrome staining, and immunofluorescence staining. For H&E staining, paraffin sections were first dewaxed in an oven at 65°C for 30 minutes, followed by xylene (twice for 10 minutes each) and a graded alcohol series (each for 10 minutes). The tissues were then stained with hematoxylin for 10 minutes followed by eosin for 1 minute. Finally, the tissues were sealed with neutral resin and directly observed under a microscope (Leica, Germany). The cross-sectional area (CSA) of muscle fibers was calculated using ImageJ software [49]. For Masson's trichrome staining, the dewaxing and rehydration processes were the same as those for H&E staining. The entire process was carried out using a commercial Masson staining kit (Solarbio, China). Fibrosis was observed under a microscope (Leica, USA), and the fibrotic areas were quantified using ImageJ software [50, 51].

### Immunofluorescence analysis

For immunofluorescence staining, as described above, the dewaxing and rehydration process was the same as that for H&E staining. Next, the surrounding tissue water was cleared, and a barrier was created around the tissue on each slide using a fluorescent pen. Subsequently, the tissue sections were blocked at room temperature with 5% BSA and 0.5% Triton-X-100 (Solarbio, China) for 1 hour, followed by overnight incubation at 4°C with diluted primary antibodies. The next day, the sections were washed three times for 5 minutes each with 1× PBST before they were incubated for 1 hour at room temperature with Alexa Fluor 488-conjugated anti-mouse (H + L) secondary antibody (1:500; Life Technologies, USA). DAPI was used for nuclear staining. Images were captured using a fluorescence microscope (Leica, Germany). All the captured images used the same microscope settings (e.g. exposure time, laser intensity, and gain).

### In vivo biosafety evaluation of W-GA nanodots

W-GA nanodots were administered orally at a dose of 100 mg/kg to C57BL/6 mice aged 6–8 weeks and weighing 18–22 grams, with PBS serving as the control. Mice were euthanized 14 days after oral administration, and their major organs were stained with H&E and Masson’s trichrome to assess biosafety.

### Statistical analysis

All experiments in this study were repeated at least three times. The data obtained from these experiments were analyzed using GraphPad Prism 9.0 (GraphPad Software, CA) and are presented as the means and standard deviations. To determine the significance of the observed differences between groups, various statistical tests were used, including the Mann–Whitney U test, Student's *t* test, one-way/two-way analysis of variance (ANOVA), or Bonferroni posthoc correction following ANOVA, depending on the distribution characteristics of the data. A *P* value less than 0.05 was considered to indicate statistical significance.

## Results

### Identification of cell subtypes in injured gastrocnemius muscles from normal and diabetic mice

**Figure 1:**
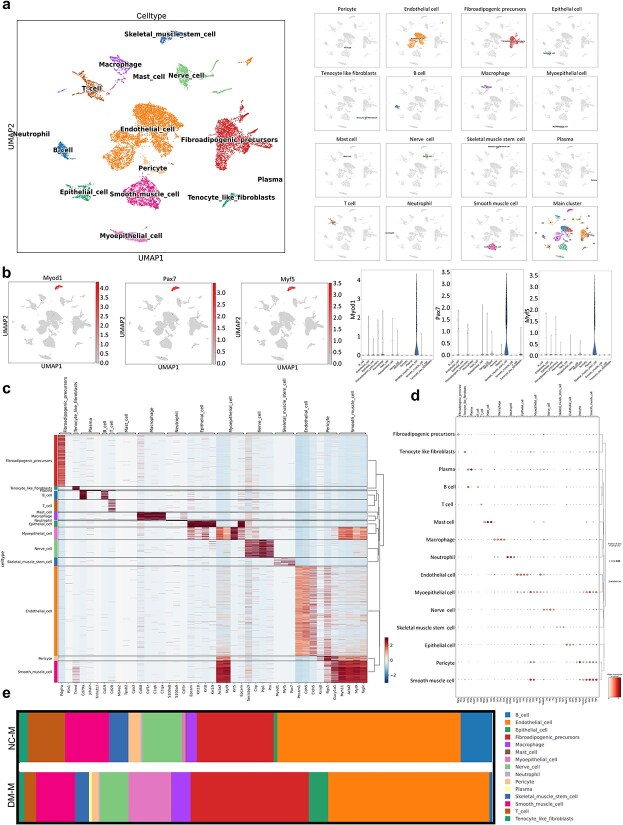
Single-cell transcriptomic analysis following acute skeletal muscle injury in diabetic mice. (**a**) Visualization of single-cell RNA sequencing data via uniform manifold approximation and projection (UMAP) to classify various cell types. Each cluster denotes a unique cell type, labeled based on its presumed biological classification. (**b**) Detailed UMAP plots displaying the distribution of specific markers across identified cell types. Each subplot represents a distinct marker, highlighting its expression pattern within the overall cellular landscape. (**c**) Heatmap showing the differential expression of selected genes among the identified cell types. Rows represent genes; columns represent cell types, with color intensity reflecting expression levels. Hierarchical clustering was applied to group similar expression patterns. (**d**) The bubble chart shows the expression distribution of selected markers across cell types. (**e**) Stacked bar charts showing the relative proportions of each cell type in the diabetic skeletal muscle injury and control skeletal muscle injury groups under two different conditions. The colors correspond to the cell types defined in the UMAP plot, showing changes in cellular composition between conditions

Single-cell RNA sequencing was performed on injured gastrocnemius muscles from both normal and diabetic mice to elucidate the cellular landscape and response to injury. Uniform manifold approximation and projection (UMAP) analysis revealed a diverse array of cell subtypes within the muscle tissue, including skeletal muscle stem cells, macrophages, mast cells, endothelial cells, fibroadipogenic progenitors, pericytes, plasma cells, epithelial cells, smooth muscle cells, tenocytes, fibroblasts, neutrophils, and myoepithelial cells (Figure 1a). The detailed expression profiles of these subtypes were further characterized through UMAP plots, heatmaps, and bubble charts (Figure 1b-d), highlighting key marker genes that define each cell population. Specifically, heatmaps and bubble charts effectively delineated the expression patterns of significant markers across the identified cell subtypes, providing insights into their functional roles during muscle repair and regeneration. Moreover, a comparative analysis of cell subtype distributions between normal and diabetic muscle samples revealed significant differences (Figure 1e). Most prominently, the proportion of skeletal muscle stem cells (MSCs) was significantly reduced in the diabetic injury group, indicating an impaired regenerative capacity in diabetic muscles. This finding underscores the detrimental impact of diabetes on muscle regeneration and highlights the importance of targeting cellular mechanisms that support muscle repair under diabetic conditions.

### In-depth bioinformatics analysis of MSC subpopulations reveals impaired stemness in diabetic muscle injury

**Figure 2:**
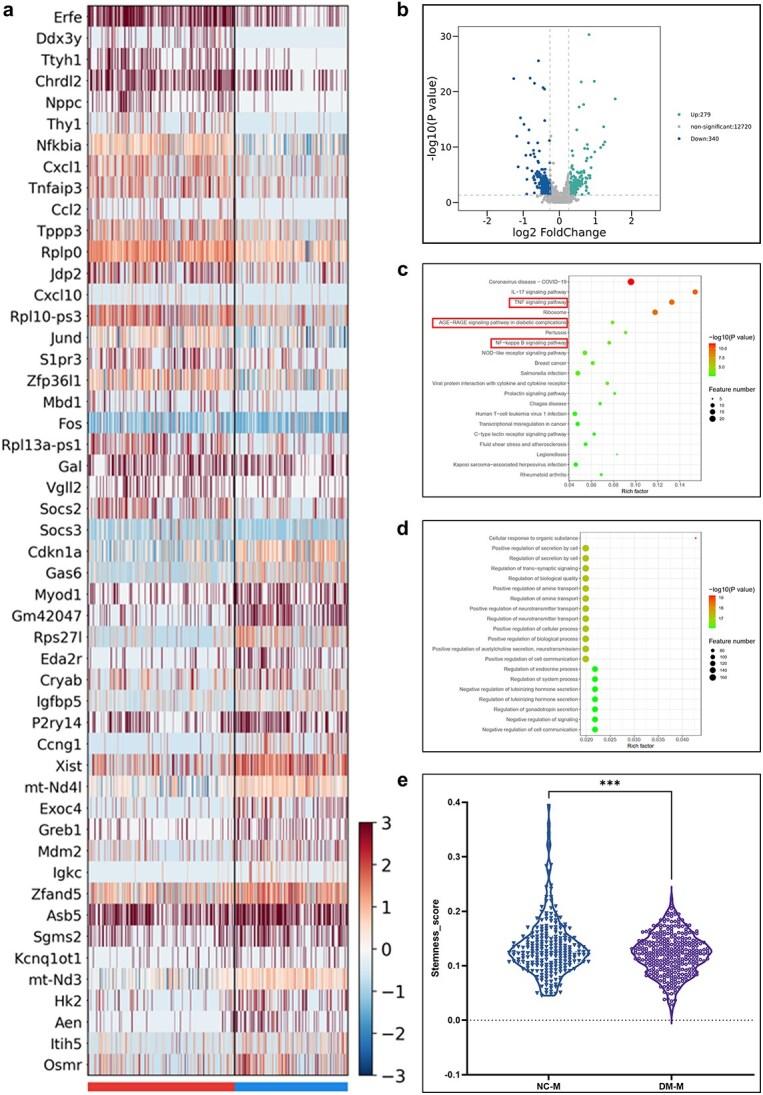
Comprehensive analysis of differential gene expression and pathway enrichment in mesenchymal stem cell (MSC) subgroups between the two conditions (**a**) Heatmap displaying DEGs in MSC subgroups between the two conditions, showing variations in gene expression levels. (**b**) Volcano plot illustrating the number of DEGs in the MSC subgroups, with significance highlighted. (**c**) KEGG pathway enrichment analysis of differentially expressed genes indicating the metabolic and signaling pathways involved. (**d**) GO pathway enrichment analysis of differentially expressed genes, showing enriched biological processes, molecular functions, and cellular components. (**e**) Violin plots comparing the expression levels of selected DEGs between nonconditioned medium (NCM) and conditioned medium (DkM), with statistical significance denoted by asterisks (^*^^*^^*^p < 0.001)

Following the identification of cell subtypes, an in-depth bioinformatics analysis was conducted, focusing on the skeletal muscle stem cell (MSC) subpopulation. Comparative gene expression analysis between MSCs from normal and diabetic injured muscles was visualized using differential gene expression heatmaps and volcano plots (Figure 2a and b). These analyses revealed distinct expression profiles, with several genes being significantly upregulated or downregulated under diabetic conditions. To further understand the biological implications of these differences, Gene Ontology (GO) and Kyoto Encyclopedia of Genes and Genomes (KEGG) pathway enrichment analyses were performed (Figure 2c and d). The results indicated significant enrichment of pathways related to inflammatory response, cellular stress, and impaired cellular repair mechanisms in diabetic MSCs. Additionally, the stemness of MSCs was assessed using a scoring system derived from gene expression patterns indicative of stem cell properties (Figure 2e). The analysis revealed a significant decrease in stemness scores in MSCs from diabetic injured muscles, indicating a compromised regenerative capacity. Based on these findings from single-cell sequencing, a novel nanoparticle material was designed and developed to enhance muscle regeneration. This innovative approach aims to address the reduced stem cell functionality observed in diabetic muscle injuries.

### Comprehensive characterization and antioxidant properties of W-GA nanodots

**Figure 3:**
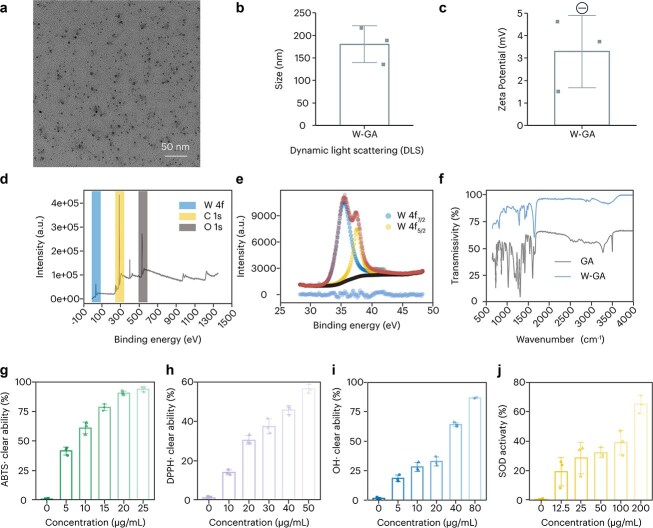
Comprehensive characterization of W-GA nanodots. (**a**) Transmission electron microscopy (TEM) image showing the morphology and dispersion of W-GA nanodots; scale bar: 50 nm. (**b**) Particle size distribution of W-GA nanodots as measured by dynamic light scattering (DLS). (**c**) Zeta potential distribution indicating the surface charge of the W-GA nanodots. (**d**) X-ray photoelectron spectroscopy (XPS) survey scan of W-GA, highlighting the presence of tungsten (W), carbon (C), and oxygen (O) peaks. (**e**) High-resolution XPS spectrum of W 4f, displaying W 4f_7/2 and W 4f_5/2 peaks. (**f**) Fourier transform infrared spectroscopy (FTIR) spectrum of GA and W-GA, demonstrating the presence of functional groups through transmissivity over a range of wavenumbers. (**g**) ABTS radical cation decolorization assay. (**h**) DPPH radical scavenging assay. (**i**) Hydroxyl radical scavenging assay. (**j**) Superoxide dismutase (SOD)-like activity assay, each showing the percentage activity relative to the control

This study comprehensively characterized W-GA nanodots, a novel nanoparticle designed for therapeutic applications(Supplementary Figure 3). The morphology and dispersion of the W-GA nanodots were visualized through transmission electron microscopy (TEM). TEM images revealed uniformly distributed nanodots with a scale bar of 50 nm, indicating well-defined size and dispersion (Figure 3a). Particle size analysis via dynamic light scattering (DLS) demonstrated that the W-GA nanodots exhibit a narrow size distribution, which is critical for their predictable behavior in biological systems (Figure 3b). Zeta potential measurements provided insights into the surface charge of the nanodots, indicating their stability in suspension as suggested by the recorded zeta potential values (Figure 3c). Chemical composition and elemental analysis were conducted using X-ray photoelectron spectroscopy (XPS). XPS survey scans confirmed the presence of tungsten (W), carbon (C), and oxygen (O) in the W-GA nanodots (Figure 3d). High-resolution XPS spectra of the W 4f region displayed distinct peaks for W 4f_7/2 and W 4f_5/2, further validating the elemental composition and oxidation state of tungsten within the nanodots (Figure 3e). Fourier transform infrared spectroscopy (FTIR) was employed to identify the functional groups on the W-GA nanodots. The FTIR spectrum revealed characteristic peaks at 3400 cm^−1^ (hydroxyl groups, -OH), 1720 cm^−1^ (carbonyl groups, C=O), and 1600 cm^−1^ (aromatic rings), which are indicative of gallic acid. Additionally, peaks at 850 cm^−1^ correspond to tungsten-oxygen bonds (W-O). These findings confirm the successful incorporation and binding of gallic acid and tungstate within the nanodots (Figure 3f). The antioxidant properties of the W-GA nanodots were evaluated using various assays to determine their potential therapeutic benefits. The ABTS radical cation decolorization assay and DPPH radical scavenging assay demonstrated significant antioxidant activity at various concentrations, indicating their ability to neutralize reactive oxygen species (Figure 3g-h). Hydroxyl and superoxide dismutase (SOD)-like activity assays further confirmed the capacity of the nanodots to scavenge hydroxyl radicals and mimic SOD activity, respectively (Figure 3i-j). These findings underscore the potential of W-GA nanodots as antioxidative agents in therapeutic settings.

### W-GA enhanced the proliferation of C2C12 cells and decreased their mortality rate in vitro

**Figure 4:**
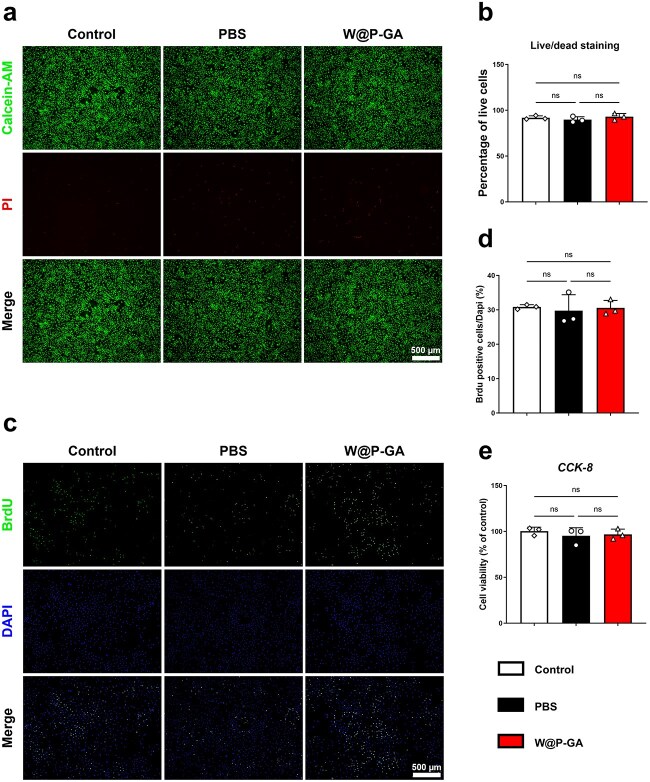
Evaluation of the biocompatibility of W-GA in vitro. (**a**) C2C12 cells cultured for 7 days in W-GA were evaluated for proliferation and cytotoxicity using live/dead staining. Scale bar = 500 μm. (**b**) Survival rate of C212 cells (*n* = 3) is shown as the mean ± standard deviation. The statistical significance of differences between treatments was determined by one-way ANOVA and the Bonferroni posthoc correction. NS: Not significant. (**c**) C2C12 cell proliferation capacity was assessed 24 hours post-treatment using BrdU incorporation. The green signal represents BrdU. Scale bar = 500 μm. (**d**) Quantification of BrdU assay data (*n* = 3). The data are presented as the mean ± standard deviation. NS: Not significant. (**e**) The cell proliferation ability of C2C12 cells in the W-GA group was further evaluated using a CCK-8 assay. The data are presented as the mean ± standard deviation. NS: Not significant

After 7 days of culturing C2C12 cells with W-GA nanodots, their survival rate was evaluated using a live/dead cell staining kit. By day 7, C2C12 cells cultured with W-GA nanodots retained their polygonal morphology, showing no morphological differences compared to cells in the PBS and control groups. Furthermore, the viability of cells in the W-GA nanodot group exceeded 90%, comparable to that in the PBS and control groups (Figure 4a and b). The BrdU assay demonstrated that the number of proliferating cells in the W-GA group continued to increase over time, with no significant differences compared to the control and PBS groups (Figure 4c and d). The CCK-8 assay revealed that the OD values were high across all three groups, with no significant differences between them, indicating good cellular compatibility of the W-GA nanodots in vitro (Figure 4e).

### W-GA reduces ROS and apoptosis, enhancing myotube differentiation in insulin-resistant C2C12 cells

**Figure 5:**
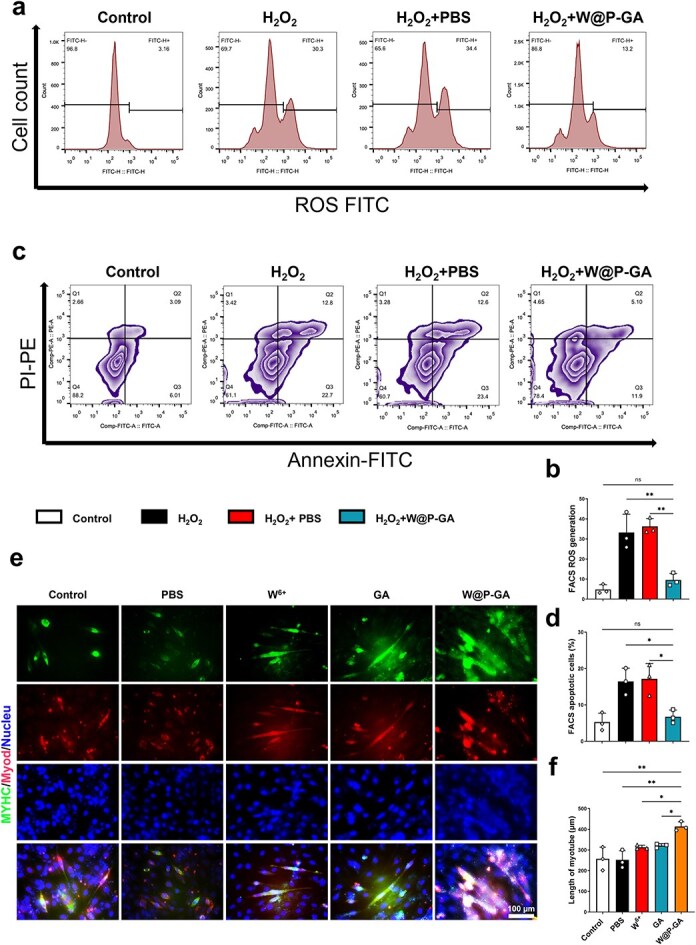
Antiapoptotic, antioxidative, and myogenic differentiation-promoting effects of W-GA. (**a**) Flow cytometry profiles showing the abundance of total C2C12 cells under various treatment conditions, along with apoptosis events in C2C12 cells under different therapeutic interventions. (**b**) Quantification of flow cytometry data for apoptotic cells (*n* = 3). The data are presented as the mean ± standard deviation. NS: Not significant, ^*^^*^p < 0.01. The statistical significance of differences between treatments was determined by one-way ANOVA and the Bonferroni posthoc correction. (**c**) Flow cytometry profiles showing the production of ROS in C2C12 cells under different treatment conditions. (**d**) Quantification of flow cytometry data for ROS production (*n* = 3). The data are presented as the mean ± standard deviation. NS: Not significant, ^*^p < 0.05. (**e**) Representative immunofluorescence image illustrating MYHC and MyoD protein expression in C2C12 myoblasts. Scale bar = 100 μm. (**f**) Quantitative analysis and intergroup comparison of myotube diameters (*n* = 3). The statistical significance of differences between treatments was determined by one-way ANOVA and the Bonferroni posthoc correction. NS: Not significant, ^*^p < 0.05, ^*^^*^p < 0.01

The fluorescent probe DCFH-DA itself is nonfluorescent. Once inside the cell, it is hydrolyzed into DCFH, which is subsequently oxidized by ROS into DCF, emitting green fluorescence. Therefore, changes in DCF fluorescence intensity can reflect dynamic variations in ROS levels. Flow cytometry was used to analyze ROS levels in each group. One day after treatment, C2C12 cells in the H_2_O_2_ group and the H_2_O_2_ + PBS group exhibited stronger DCF fluorescence, indicative of increased ROS levels. Conversely, C2C12 cells in the H_2_O_2_ + W-GA group showed weaker DCF fluorescence, suggesting reduced ROS levels, indicating that W-GA can inhibit ROS production in insulin-resistant C2C12 cells in vitro (Figure 5a and b). Phosphatidylserine (PS) exposure on the cell membrane is a well-established indicator of early apoptosis. Typically, live cells bind to Annexin V and PS but not to propidium iodide (PI). Consequently, dual staining was performed on C2C12 samples from the control, H_2_O_2_, H_2_O_2_ + PBS, and H_2_O_2_ + W-GA groups under insulin-resistant conditions. Flow cytometry results indicated that W-GA significantly reduced H_2_O_2_-induced apoptosis, from approximately 12% to approximately 5% (Figure 5c-d). These findings suggest that W-GA alleviates oxidative stress-induced apoptosis, thereby providing cellular protection and indicating its potential therapeutic role in tissue damage. Additionally, W-GA treatment significantly influenced myotube differentiation in C2C12 myotubes, leading to a marked increase in myotube size (Figure 5e and f).

### W-GA enhances the repair of skeletal muscle injuries in diabetic mice in vivo

Prior to modeling, the mice were fed a high-fat (high-sugar) diet for 12 weeks, resulting in significant weight gain and the induction of insulin resistance, followed by intraperitoneal injections of low-dose STZ for five consecutive days. After the injections, blood glucose levels were measured every 3 days. A fasting blood glucose level of ≥11 mmol/L indicated successful model establishment (Supplementary Figure 1).

**Figure 6:**
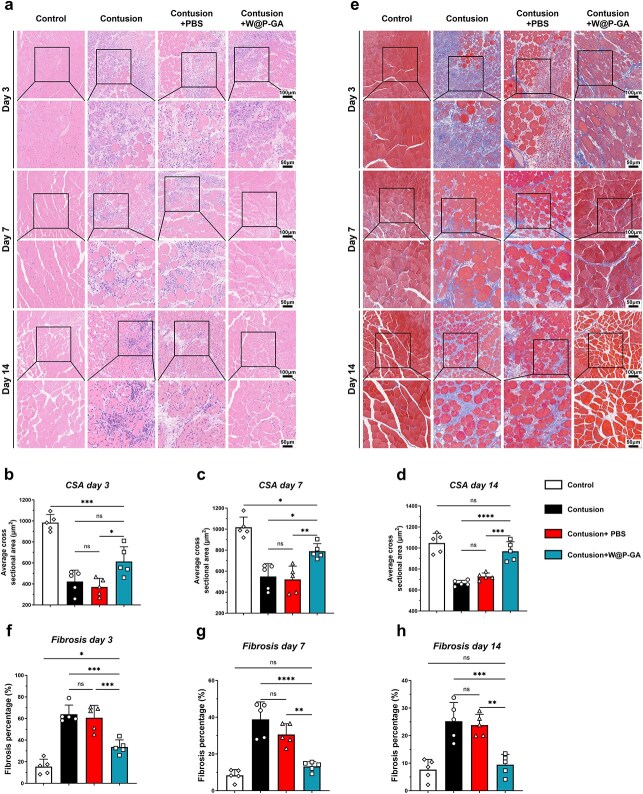
W-GA suppressed fibrosis in diabetic injured muscle in vivo and enhanced muscle regeneration. (**a**) Representative HE staining images of injured muscles on days 3, 7, and 14 among the different groups. Scale = 100 or 50 μm. (**b**-**d**) Comparisons of muscle fiber cross-sectional areas (CSAs) among the different groups on days 3, 7, and 14 (*n* = 5). NS: Not significant; ^*^p < 0.05, ^*^^*^p < 0.01, ^*^^*^^*^p < 0.001. (**e**) Representative Masson's trichrome staining images of injured muscles at days 3, 7, and 14 among the different groups. (f-h) Comparisons of muscle fibrosis percentage among the different groups on days 3, 7, and 14 (*n* = 5). NS: Not significant; ^*^p < 0.05, ^*^^*^p < 0.01, ^*^^*^^*^p < 0.001, ^*^^*^^*^^*^p < 0.0001

On days 3 and 7 postinjury, HE staining of muscle tissues revealed increased cellular infiltration and extensive necrotic fibers in the contusion and contusion+PBS groups. Compared with the contusion and contusion+PBS groups, the contusion+W-GA group exhibited significantly less necrosis and infiltration, indicating improved conditions. By day 14 postinjury, ongoing cellular infiltration was observed in the contusion and contusion+PBS groups, while muscle tissues in the contusion+W-GA group had recovered to control group levels. Additionally, the cross-sectional area (CSA) of muscle fibers in the contusion+W-GA group significantly improved on days 3, 7, and 14 (Figure 6a-d). Masson staining of muscles on days 3, 7, and 14 revealed significant interstitial fibrosis in the contusion and contusion+PBS groups, which was substantially reduced in the contusion+W-GA group (Figure 6e-h).

**Figure 7:**
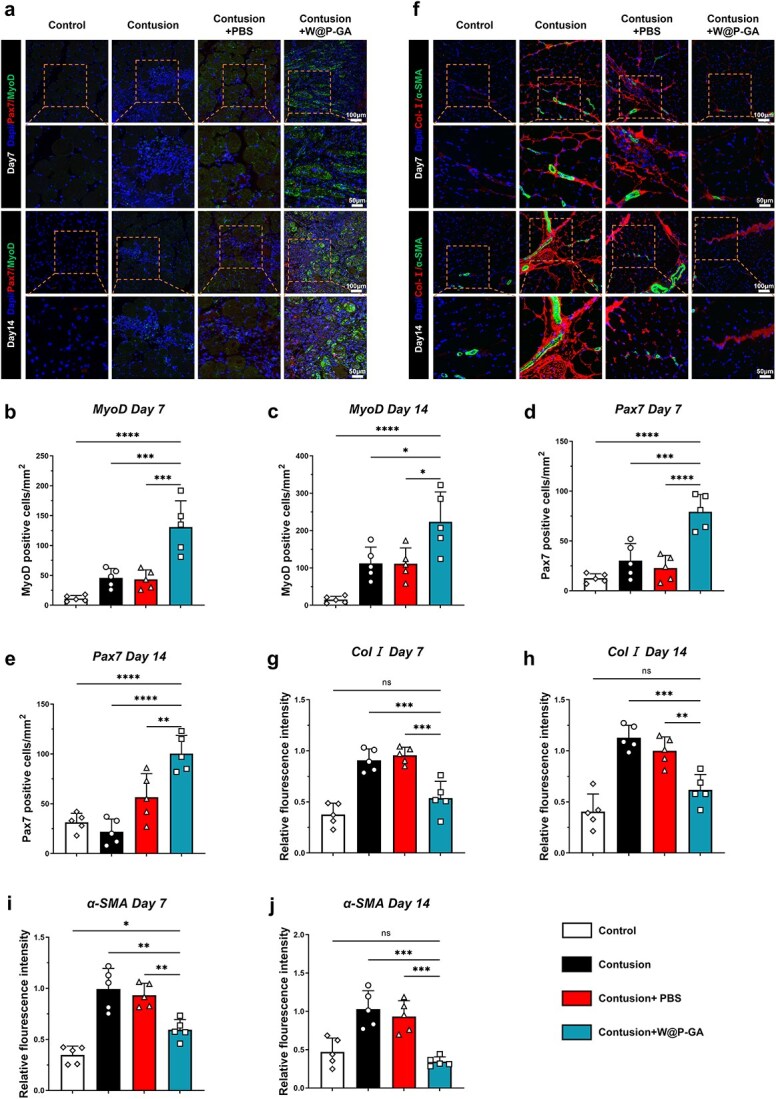
Immunofluorescence staining of skeletal muscle in diabetic mice. (**a**) On days 7 and 14, immunofluorescence was used to detect the relative quantity and distribution of Pax7 and MyoD in the different groups. Scale = 100 or 50 μm. (**b**-**e**) Quantitative analysis of Pax7- and MyoD-positive cells on days 7 and 14 following various treatments (*n* = 5). The data are presented as the means ± standard deviations. Statistical significance was determined by one-way ANOVA and Bonferroni posthoc analysis. Significance levels are indicated as NS: Not significant; ^*^p < 0.05, ^*^^*^^*^p < 0.001, ^*^^*^^*^^*^p < 0.0001. (**f**) On days 7 and 14, immunofluorescence was used to assess the relative expression and distribution of type I collagen and α-SMA in the different groups. Scale bar = 100 or 50 μm. (**i**-**j**) Relative fluorescence intensity of type I and III collagen in the various groups on days 7 and 14 (*n* = 5). The statistical significance of differences among treatments was determined using one-way ANOVA and Bonferroni posthoc correction. NS: Not significant, ^*^p < 0.05, ^*^^*^p < 0.01, and ^*^^*^^*^p < 0.001

Immunofluorescence staining was employed to assess the spatial expression of bioactive proteins at various time points. Compared with the contusion and contusion+PBS groups, the contusion+W-GA group exhibited a significant increase in newly formed muscle fibers (MyoD+Pax7+) on days 7 and 14, suggesting that W-GA treatment enhances muscle fiber regeneration (Figure 7a-e). On days 7 and 14 postcontusion, staining for tenascin and collagen (α-SMA + Col I+) revealed extensive fibrosis and disorganized tissue arrangement in the contusion and contusion+PBS groups. In contrast, the contusion+W-GA group displayed an orderly tissue arrangement, larger muscle fibers, and reduced collagen deposition, indicating that W-GA treatment significantly reduced skeletal muscle fibrosis (Figure 7f-j).

These findings comprehensively confirm the therapeutic effects of W-GA on damaged muscles at various stages in diabetic mice, demonstrating superior outcomes in fibrosis reversal, reduced cellular infiltration, and enhanced muscle regeneration in the contusion+W-GA group.

### W-GA enhances functional recovery following diabetic skeletal muscle injury

**Figure 8:**
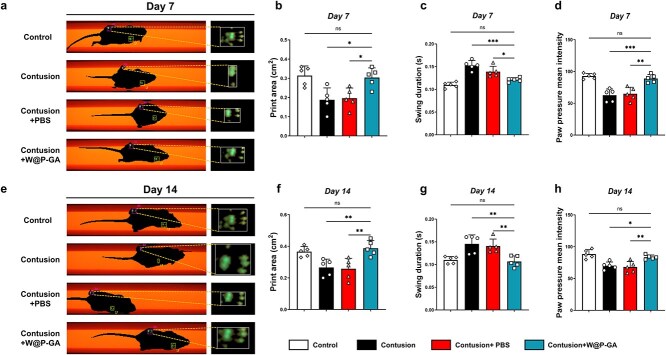
Gait test of diabetic skeletal muscle injury mice. (**a**) Maximum contact area of the right hindlimb 7 days after surgical intervention. (**b**-**d**) Quantitative analysis of three gait parameters of the hindlimb 7 days after surgical intervention, including average paw pressure, paw print area, and swing time (*n* = 5). The data are presented as the mean ± standard deviation. The statistical significance of differences between treatments was determined using one-way ANOVA and the Bonferroni posthoc correction. NS: Not significant, ^*^p < 0.05, ^*^^*^p < 0.01, and ^*^^*^^*^p < 0.001. (**e**) Maximum contact area of the right hindlimb 14 days after surgical intervention. (**f**-**h**) Quantitative analysis of three gait parameters of the hindlimb 7 days after surgical intervention, including average paw pressure, paw print area, and swing time (*n* = 5). The data are presented as the mean ± standard deviation. The statistical significance of differences between treatments was determined using one-way ANOVA and the Bonferroni posthoc correction. NS: Not significant, ^*^p < 0.05 and ^*^^*^p < 0.01

To evaluate muscle function recovery after skeletal muscle injury in diabetic mice, we analyzed the walking patterns of the blunt contusion, blunt contusion+PBS, blunt contusion+W-GA, and control groups at 7 and 14 days postinjury. Walking patterns more closely resembling those of the control group were considered positive indicators of functional recovery. The CatWalk XT gait analysis system (Noldus, the Netherlands) was employed to monitor the locomotor performance of each mouse. Parameters such as maximum plantar contact area, average PWCF, and swing phase duration were used to assess gastrocnemius muscle functional recovery. At both postinjury time points, the blunt contusion+W-GA group demonstrated a significantly greater maximum contact area and average PWCF on the injured side compared to the blunt contusion and blunt contusion+PBS groups (Figure 8a-c, e-g). Across all behavioral metrics, the W-GA group outperformed the other groups and closely matched the control group, suggesting that W-GA facilitated muscle function recovery after skeletal muscle injury in diabetic mice.

### W-GA demonstrates good biocompatibility in vivo

Histological evaluation highlighted the excellent biosafety of W-GA upon introduction into living organisms. On days 7 and 14 postinjection, vital organs, including the heart, liver, spleen, lungs, and kidneys, showed no significant changes compared to the control group (Supplementary Figure 2a). Additionally, Masson's trichrome staining of heart, liver, spleen, lung, and kidney tissues revealed no collagen accumulation or perivascular lymphocyte clustering (Supplementary Figure 2B). These histological findings confirmed the in vivo biocompatibility and safety of W-GA.

## Discussion

### Implications of W-GA nanodots in diabetic myopathy

**Figure 9:**
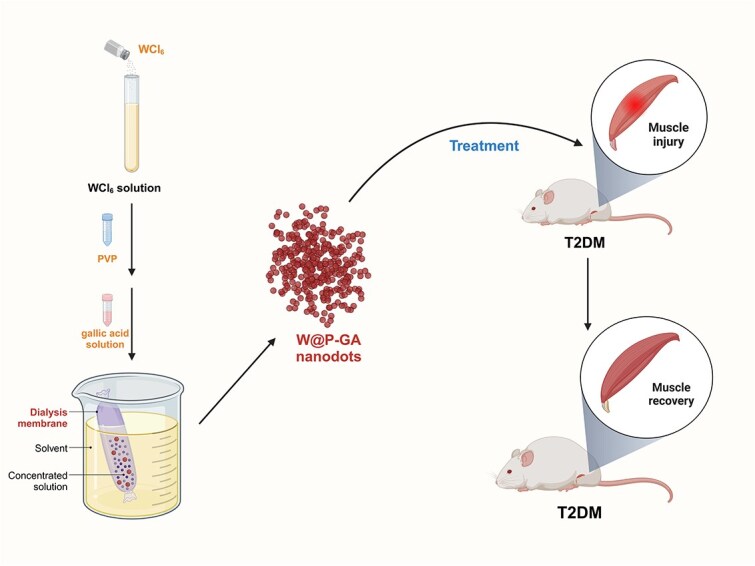
Schematic diagram showing how W-GA nanodots promote the repair of diabetic skeletal muscle injury

The results of our investigation into the therapeutic potential of W-GA nanodots for diabetic muscle injury hold significant implications for the clinical management of diabetic myopathy. (Figure 9) Diabetic myopathy, characterized by impaired muscle regeneration and chronic inflammation, presents a substantial challenge in diabetes care, exacerbating the progression of T2D and its associated complications [15]. The pathophysiological landscape of diabetic myopathy involves a complex interplay of metabolic derangements, including insulin resistance, chronic low-grade inflammation, and oxidative stress, culminating in progressive muscle dysfunction and impaired regenerative capacity [52].

Our study introduces W-GA nanodots as a novel therapeutic strategy, leveraging the anti-inflammatory and antioxidative properties of gallic acid along with the multifunctional characteristics of tungstate to ameliorate muscle damage in diabetic contexts. These nanodots significantly enhanced muscle fiber regeneration, reduced oxidative stress, and diminished inflammatory infiltration in the muscle tissue of diabetic mice.

### Enhancing muscle regeneration and function

The muscle-regenerative potential of W-GA nanodots was demonstrated by their impact on C2C12 cells. In diabetic muscles, the regenerative capacity of MSCs is notably compromised, a phenomenon addressed by our treatment through the improvement of the cellular microenvironment, thereby enhancing the regenerative and proliferative capabilities of these cells. Histological analyses further confirmed muscle tissue regeneration, as evidenced by increased muscle fiber size and reduced fibrosis in the treated groups compared to the control group.

The functional implications of these findings were further substantiated by gait analysis, which demonstrated improved locomotor performance in mice treated with W-GA. This recovery of muscle function is particularly crucial for improving the quality of life in diabetic patients, as muscle weakness and poor regeneration often lead to physical disability [53].

### Addressing oxidative stress and inflammation

A critical aspect of our findings is the role of W-GA nanodots in mitigating oxidative stress and inflammation, two key contributors to muscle damage in diabetes. By reducing ROS production and limiting oxidative stress-induced apoptosis, the nanodots create a more favorable environment for muscle repair and regeneration. This antioxidative action, combined with the anti-inflammatory effects observed through reduced inflammatory cell infiltration and cytokine expression, underscores the dual therapeutic potential of W-GA nanodots.

### Clinical relevance and future directions

Although our results are promising, transitioning from preclinical success to clinical application requires careful consideration of the pharmacokinetics, biodistribution, and long-term safety of the nanodots. The favorable biosafety profile observed in our histological evaluations provides preliminary confirmation of the feasibility of clinical application. However, further studies are necessary to optimize dosing regimens, understand long-term effects, and explore potential combination therapies that might enhance the efficacy of W-GA nanodots.

## Conclusions

In conclusion, our study not only underscores the therapeutic potential of W-GA nanodots for treating diabetic muscle injuries but also paves the way for the application of similar nanotechnology-based approaches to other diabetic complications. Future investigations should focus on detailed mechanistic studies and clinical trials to fully establish the utility and safety of this innovative treatment strategy within the broader context of diabetes management.

## Supplementary Material

Sup-8_21_tkae059

## Abbreviations

GA: Gallic acid; W6+: tungstate; T2D: Type 2 diabetes mellitus; ROS: Reactive oxygen species; MSCs: Muscle stem cells; PVP: Polyvinylpyrrolidone; SOD: Superoxide dismutase; XPS: X-ray photoelectron spectroscopy; PBS: Phosphate-buffered saline; PI: Propidium iodide; IPGTT: Intraperitoneal glucose tolerance test; GM: Gastrocnemius muscle; GO: Gene Ontology; KEGG: Kyoto Encyclopedia of Genes and Genomes; DEGs: Differentially expressed genes; FDR: False discovery rate; PFA: Paraformaldehyde; H&E: Hematoxylin and eosin; CSA: The cross-sectional area; UMAP: Uniform manifold approximation and projection; TEM: Transmission electron microscopy; DLS: Dynamic light scattering.
