# Supplementary material for: Enhancing diabetic muscle repair through W-GA nanodots: a nanomedicinal approach to ameliorate myopathy in type 2 diabetes
Source: Burns Trauma. 2025 Jan 24;13:tkae059. doi: 10.1093/burnst/tkae059 (PMC11757907; doi:10.1093/burnst/tkae059)
Supplement: Sup-8_21_tkae059 [file sup-8_21_tkae059.docx]

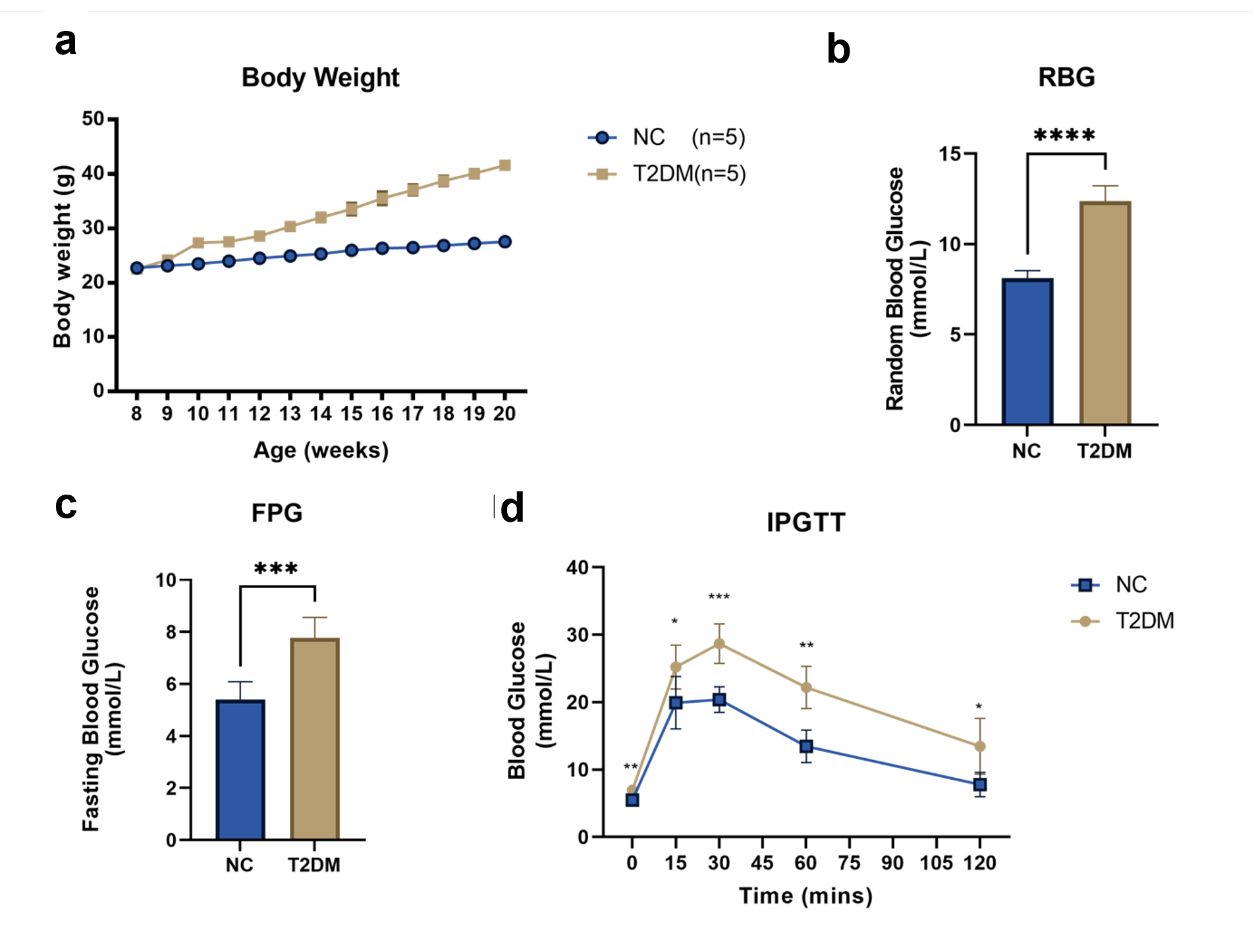


Supplementary Figure 1. Effects of Type 2 Diabetes on Body Weight and Glucose Levels in a Mouse Model. (a) displays the body weight progression in grams of mice from age 8 to 20 weeks, comparing normal control (NC) mice with type 2 diabetes mellitus (T2DM) mice. (b) illustrates random blood glucose levels (RBG) measured in mmol/L for both groups, showing significantly higher glucose levels in T2DM mice compared to NC mice (****p<0.0001). (c) presents fasting plasma glucose (FPG) levels, where T2DM mice also exhibited significantly elevated glucose levels (***p<0.001). (d) shows the results of an intraperitoneal glucose tolerance test (IPGTT), with glucose measurements at various time points over 120 minutes, indicating impaired glucose tolerance in T2DM mice compared to NC mice. Significant differences at specific time points are marked (*p<0.05, **p<0.01, ***p<0.001).


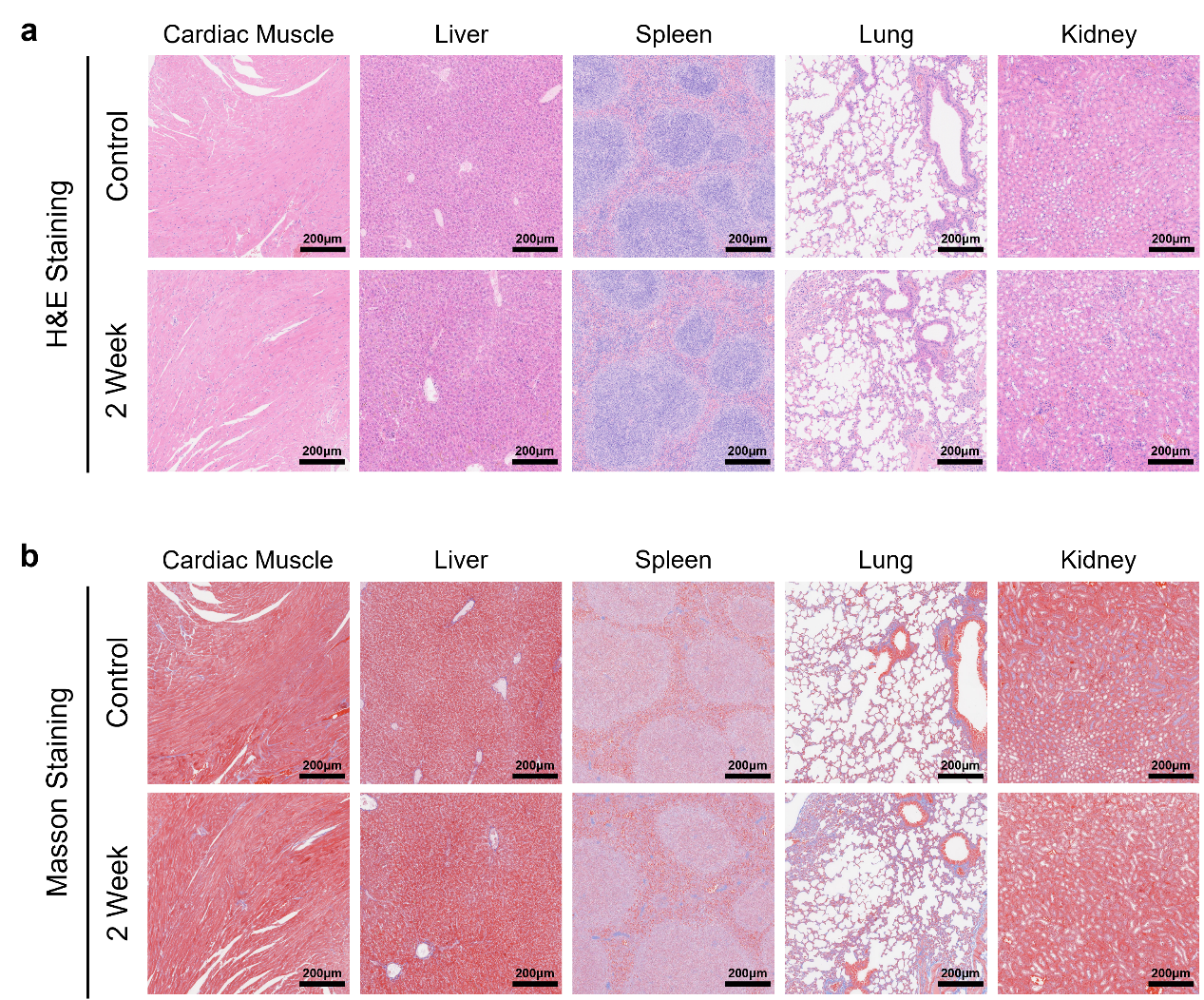


Supplementary Figure 2. Two-Week Post-Injection Analysis of W-GA nanodots Biotoxicity in vivo. (a) H&E staining of cardiac, hepatic, splenic, pulmonary, and renal tissues. Scale bar = 200 µm. (b) Masson staining of cardiac, hepatic, splenic, pulmonary, and renal tissues. Scale bar = 200 µm.


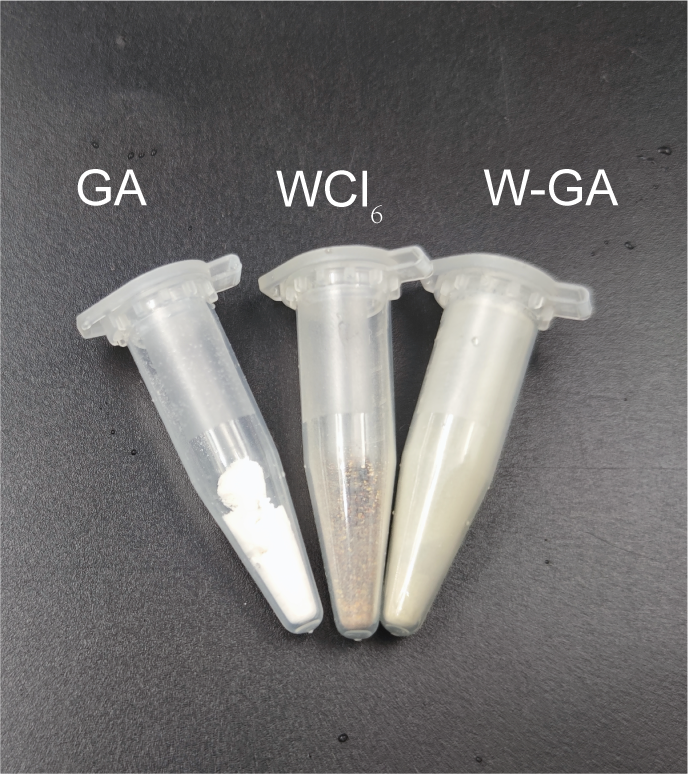


Supplementary Figure 3. Macroscopic Display of the GA, WCL_6_ (W^6+^), W-GA.
